# Supplementary material for: Four Types of TiO2 Reduced the Growth of Selected Lactic Acid Bacteria Strains
Source: Foods. 2021 Apr 25;10(5):939. doi: 10.3390/foods10050939 (PMC8146636; doi:10.3390/foods10050939)
Supplement: Supplementary file 1 [file foods-10-00939-s001.zip › Supplementary Material/Figure. S18.docx]

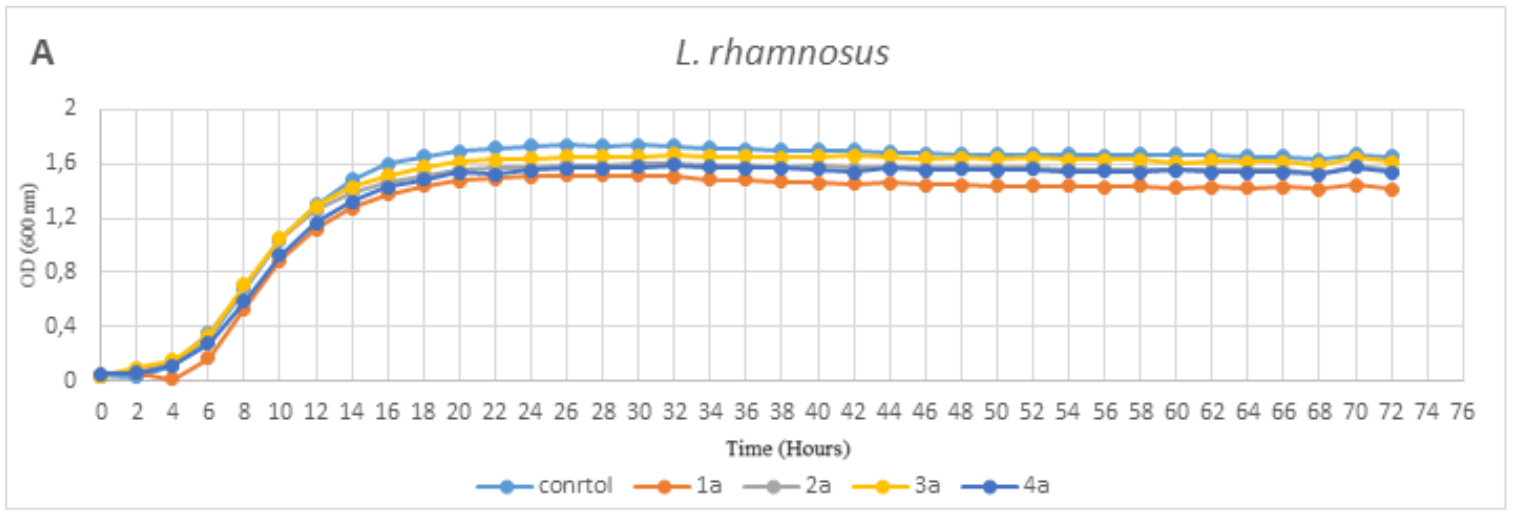

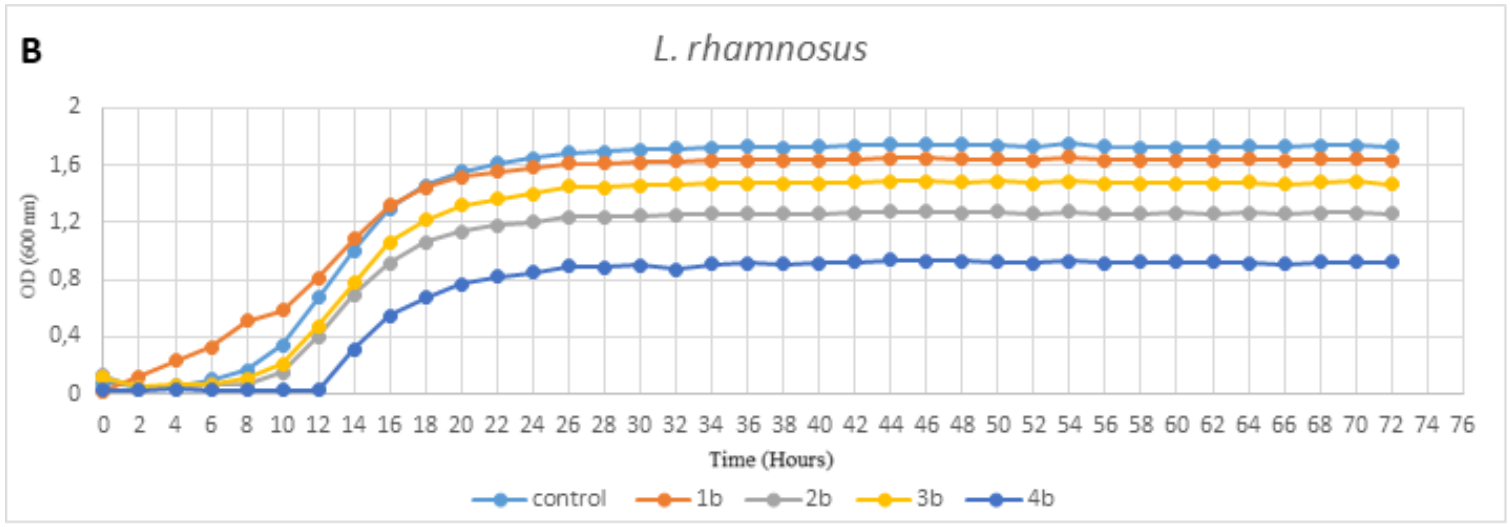


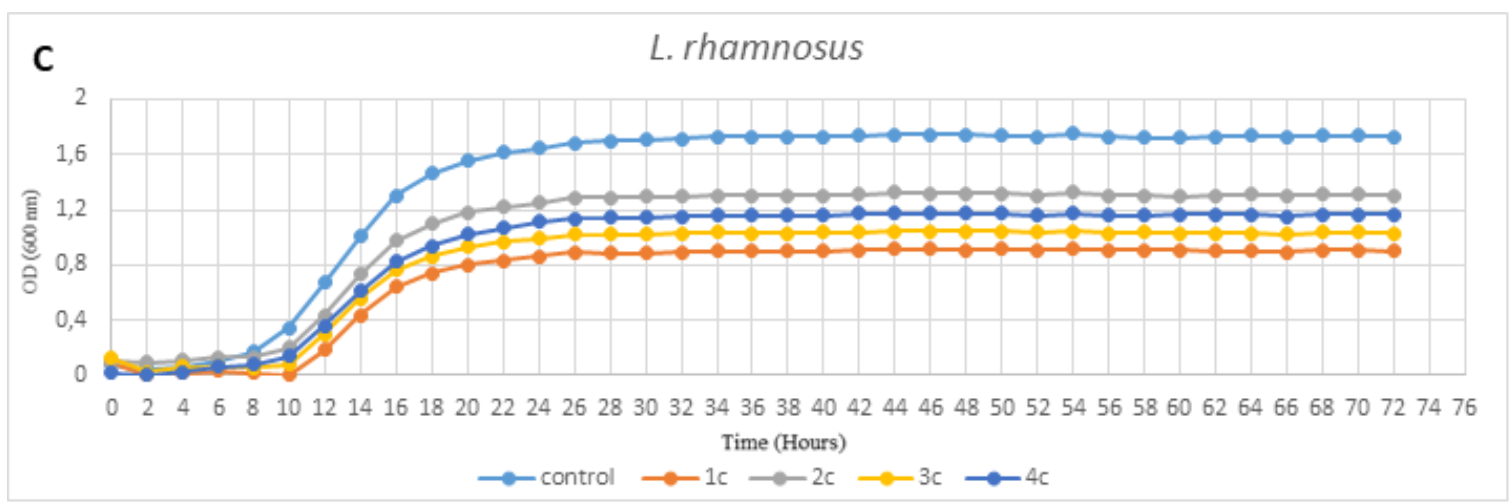


**Figure. S18.** Growth of selected bacteria after application of four types of TiO_2_ at the concentration of 600 mg/L; E171 (No. 1, 2, 3), TiO_2_ NPs (No. 4).
