# Supplementary material for: Four Types of TiO2 Reduced the Growth of Selected Lactic Acid Bacteria Strains
Source: Foods. 2021 Apr 25;10(5):939. doi: 10.3390/foods10050939 (PMC8146636; doi:10.3390/foods10050939)
Supplement: Supplementary file 1 [file foods-10-00939-s001.zip › Supplementary Material/Figure. S8.docx]

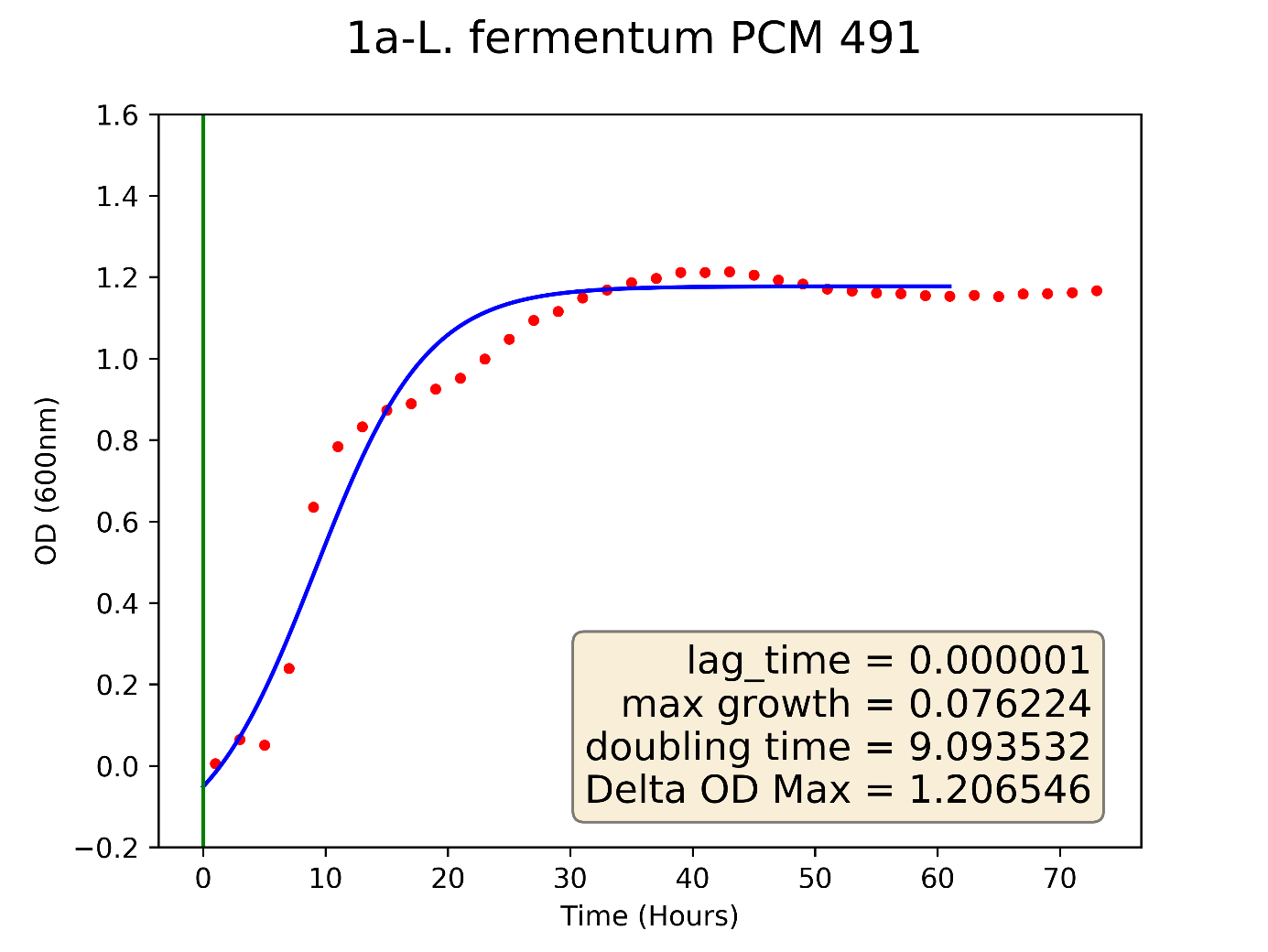


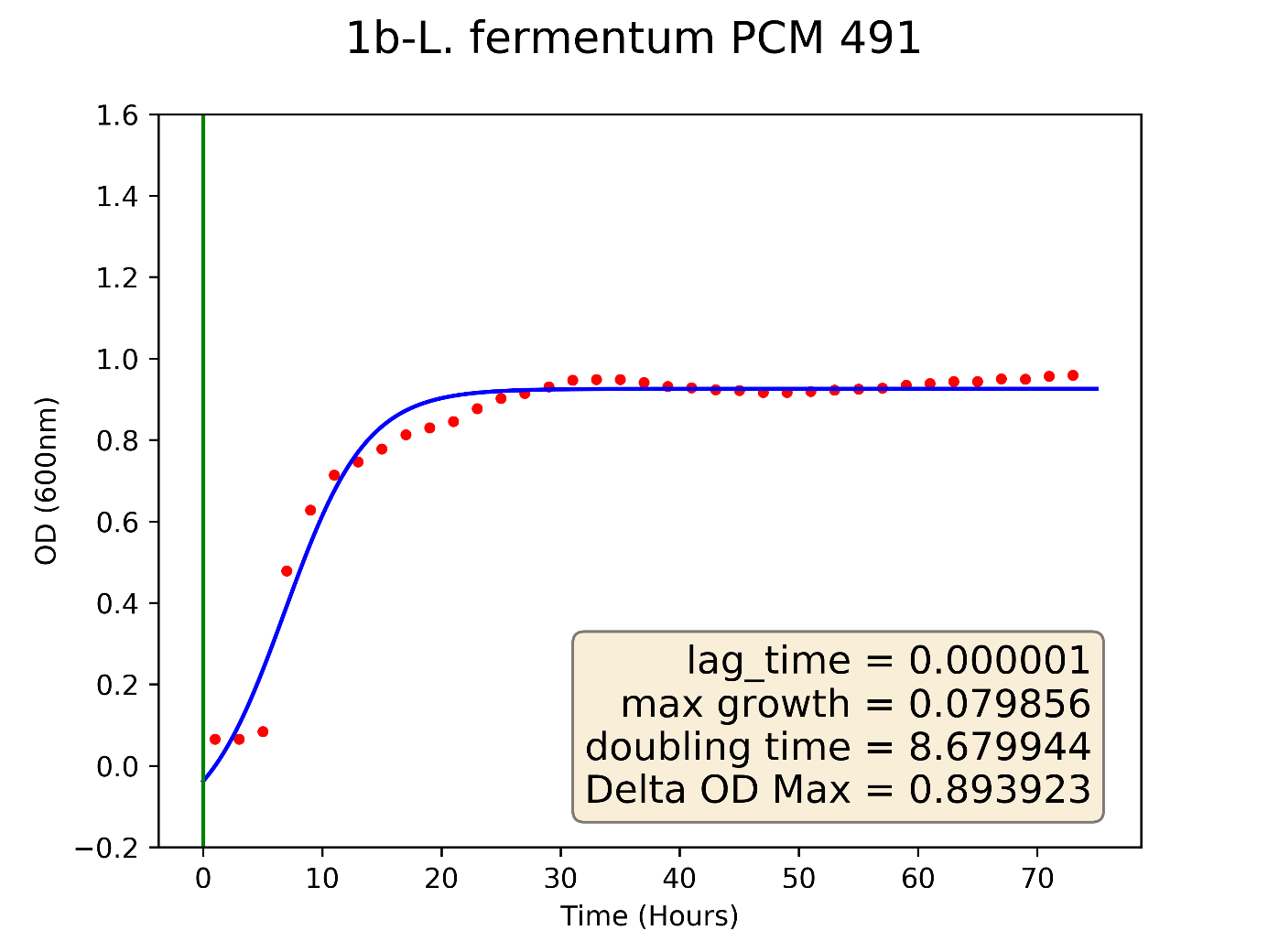


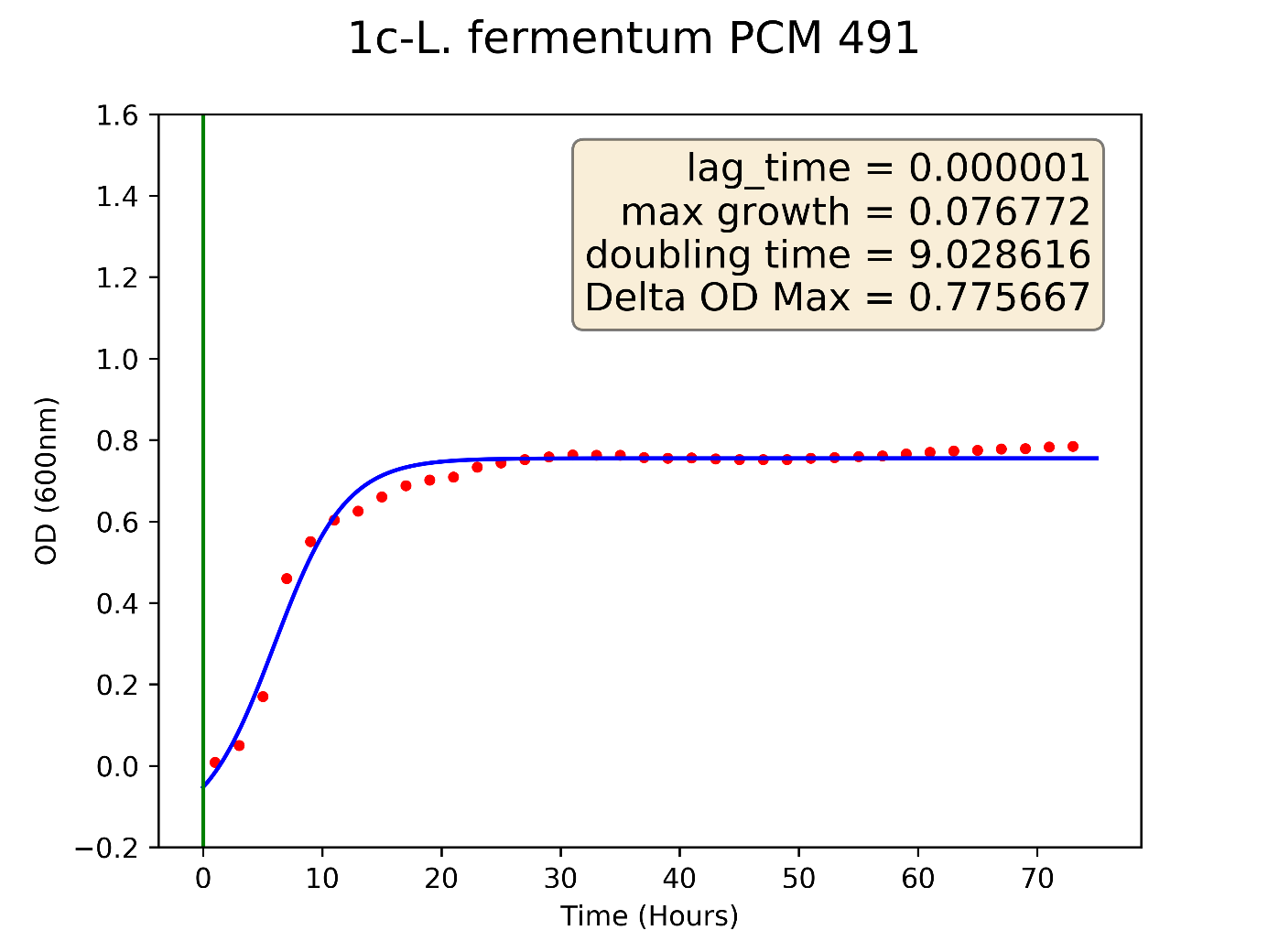


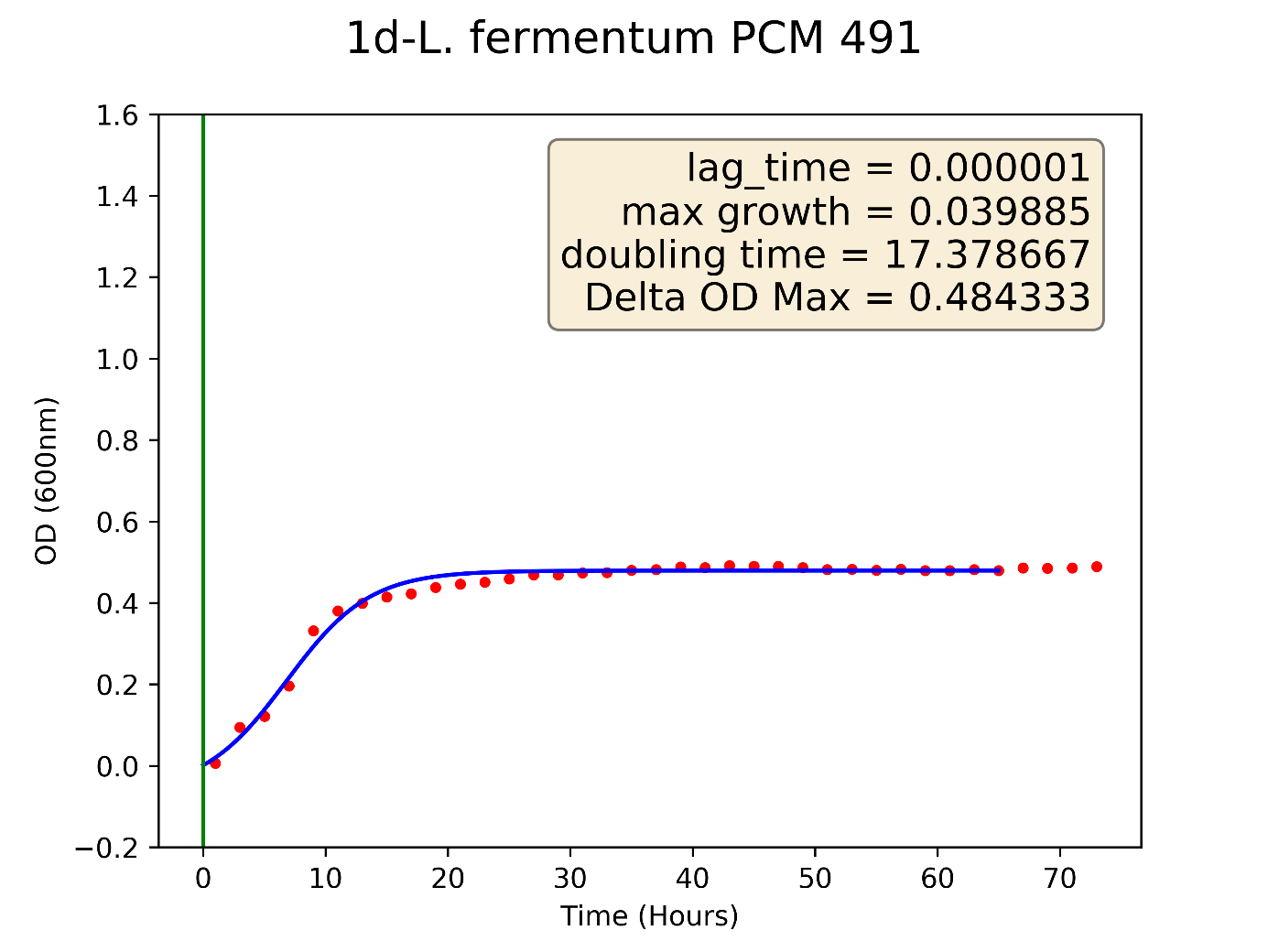


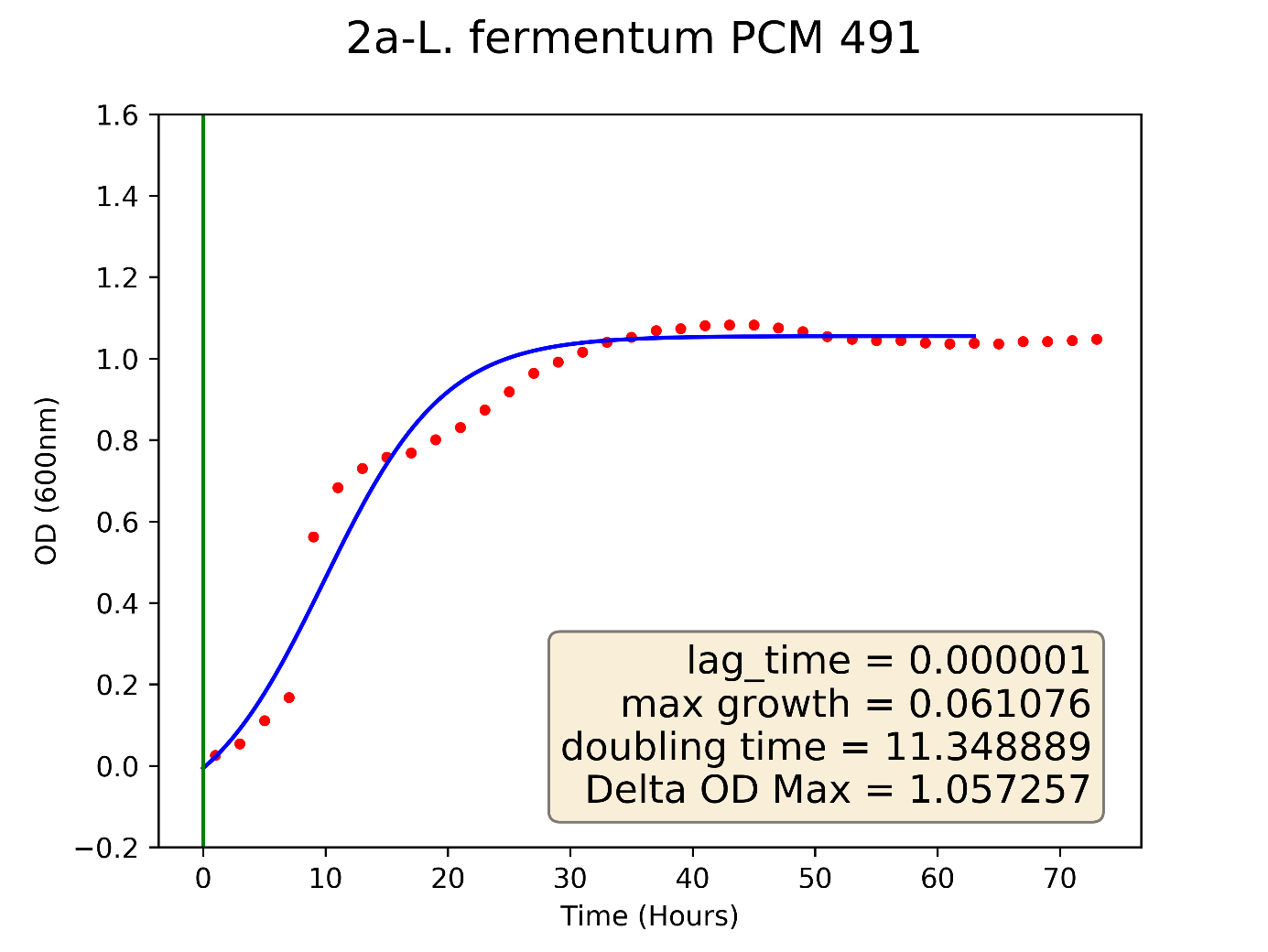


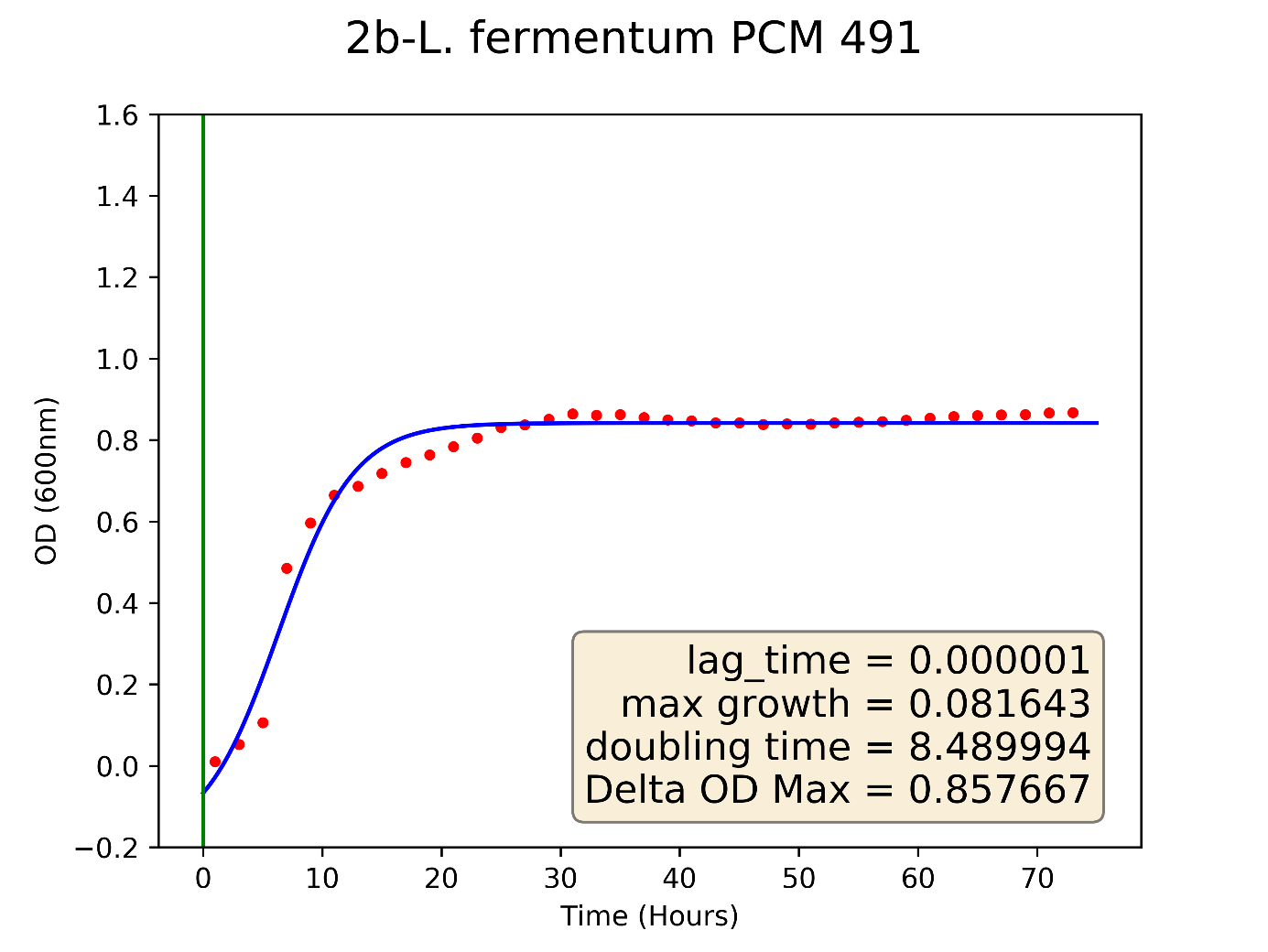


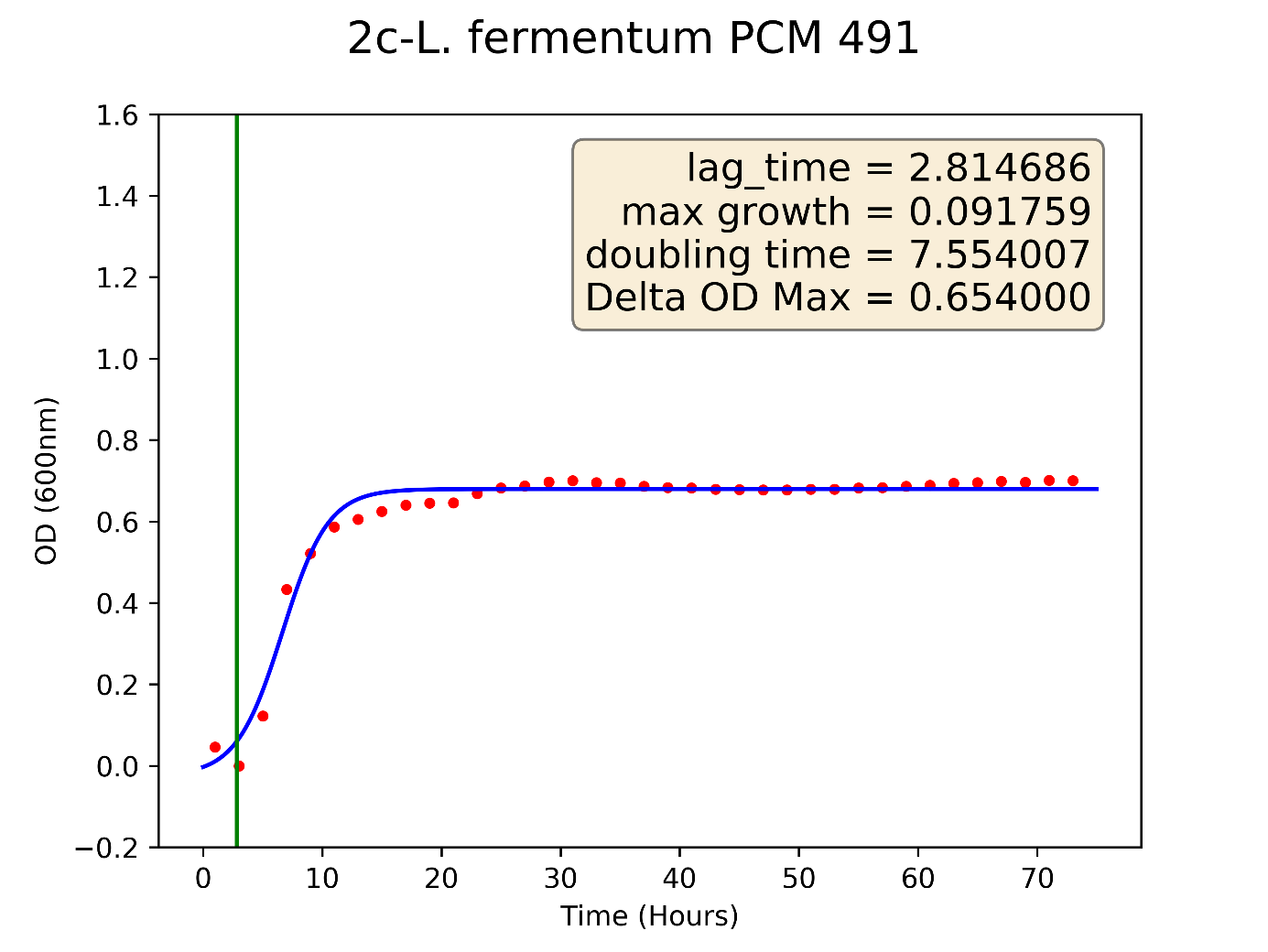


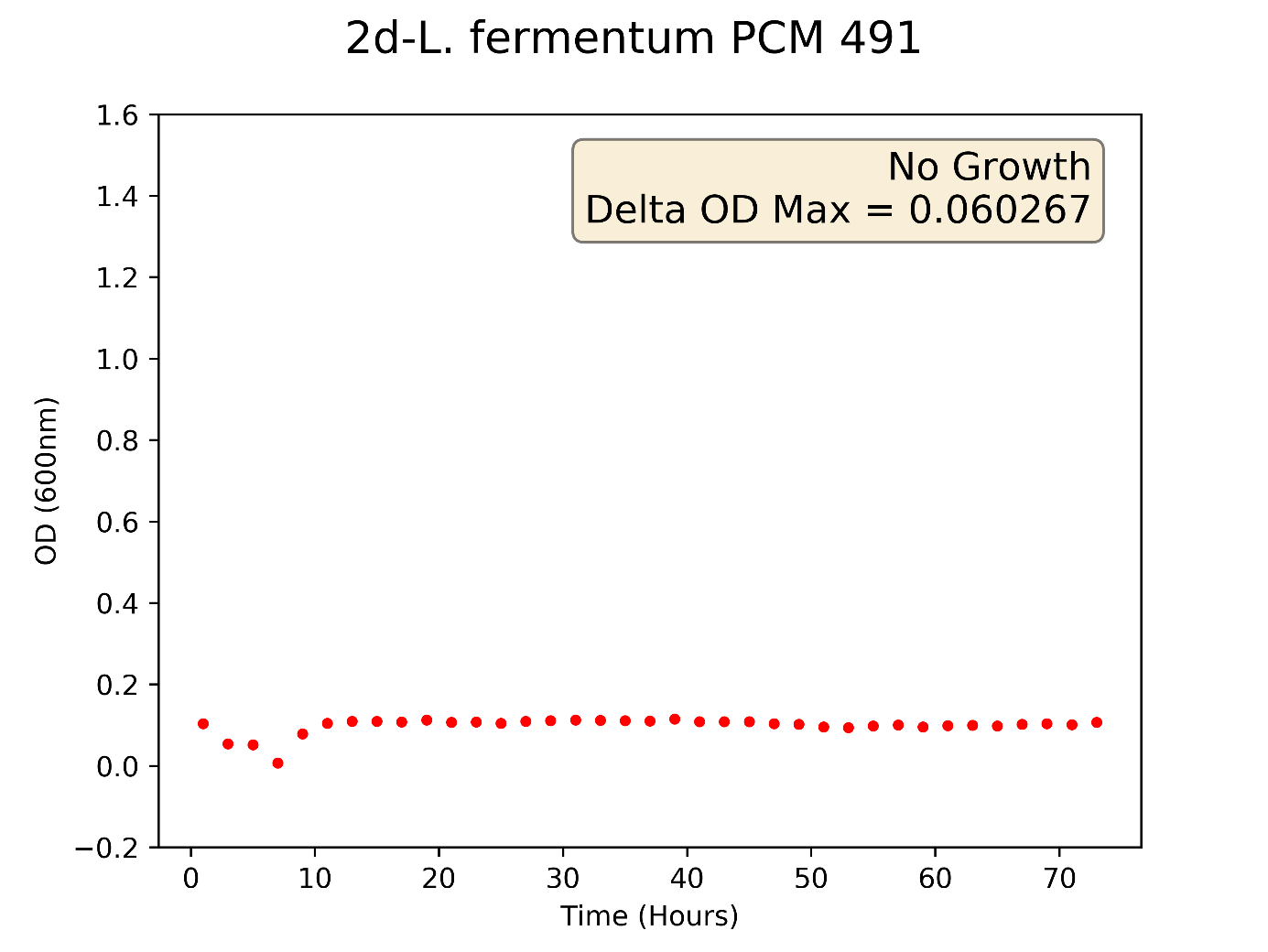


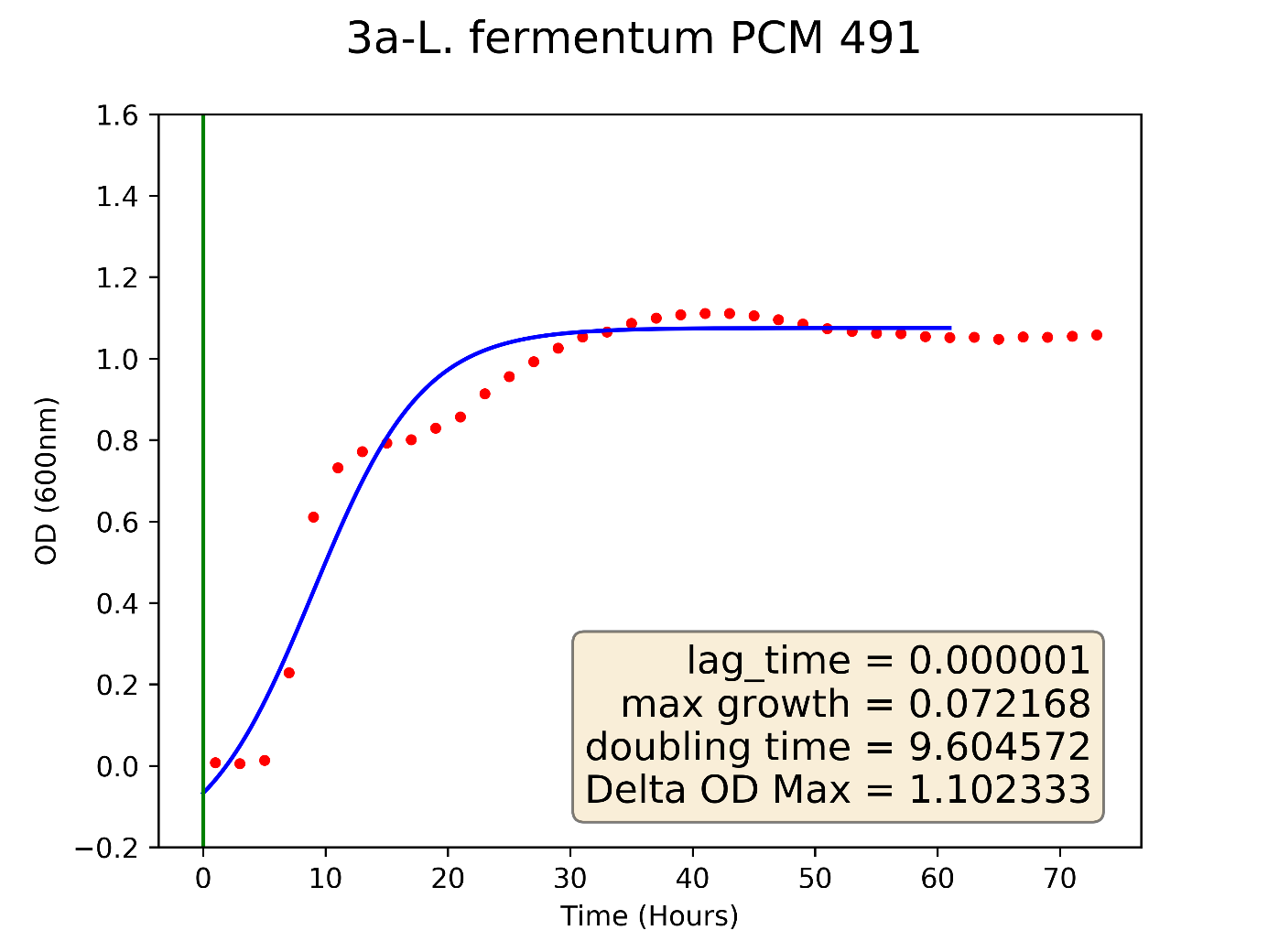


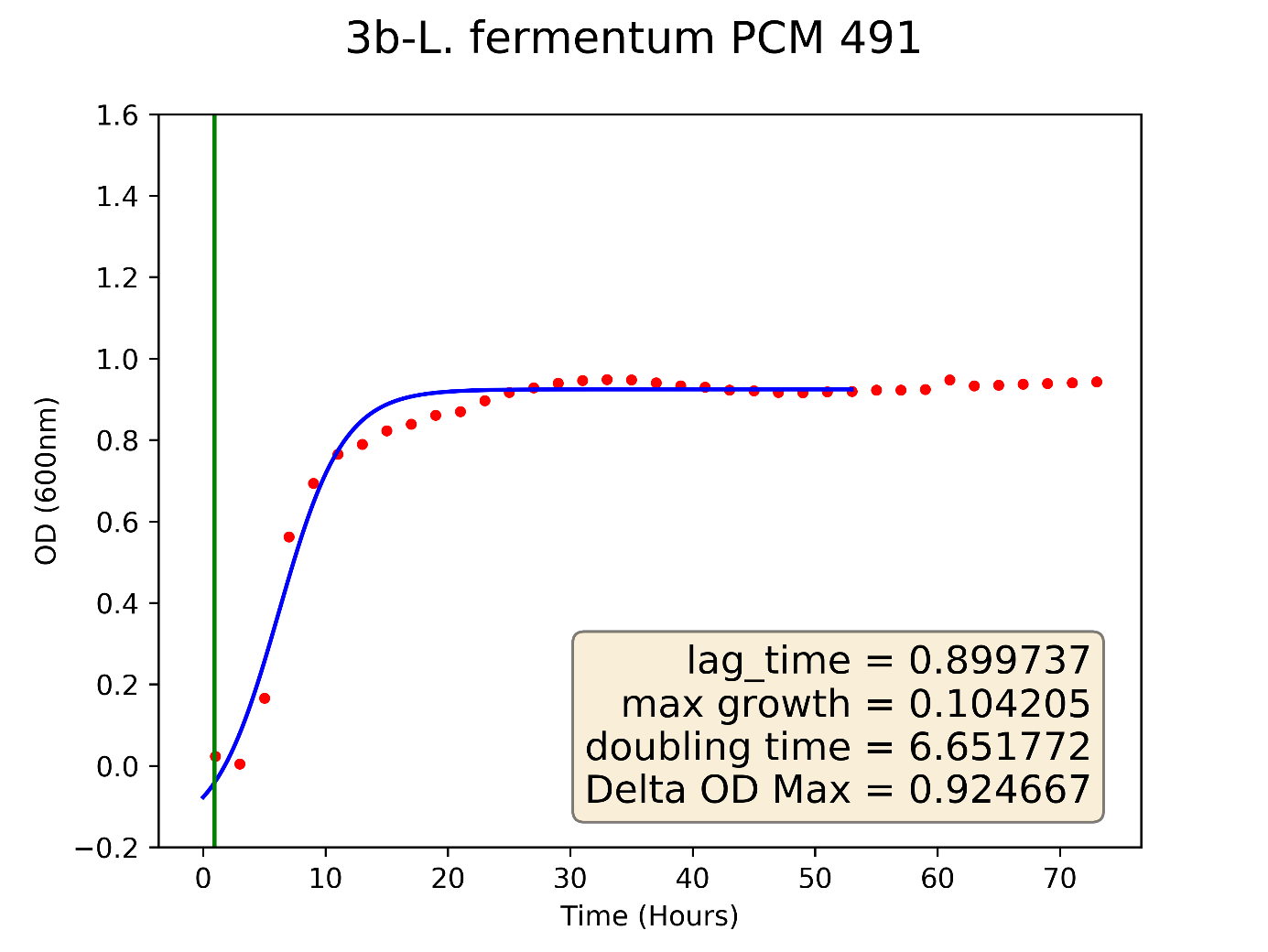


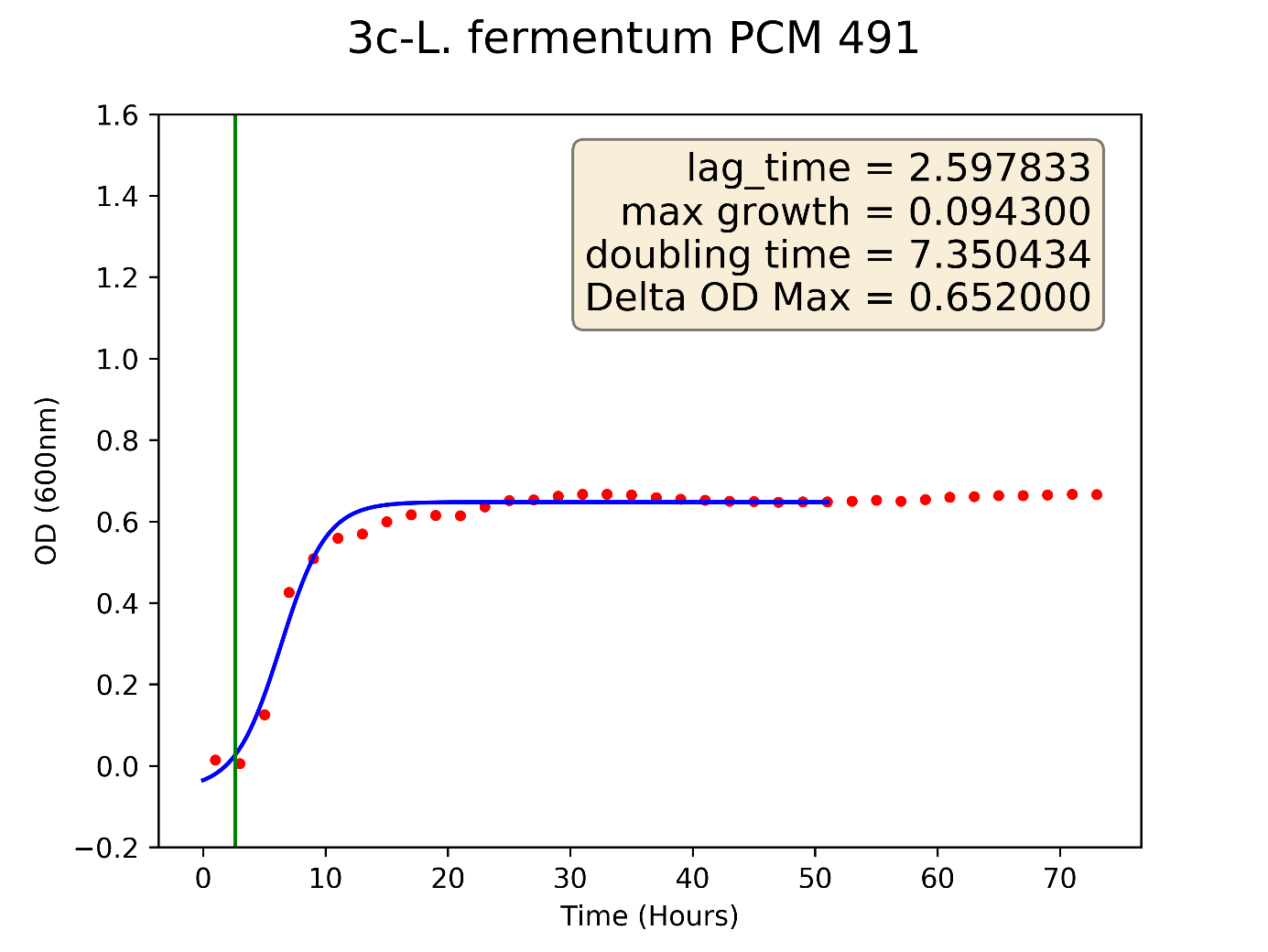


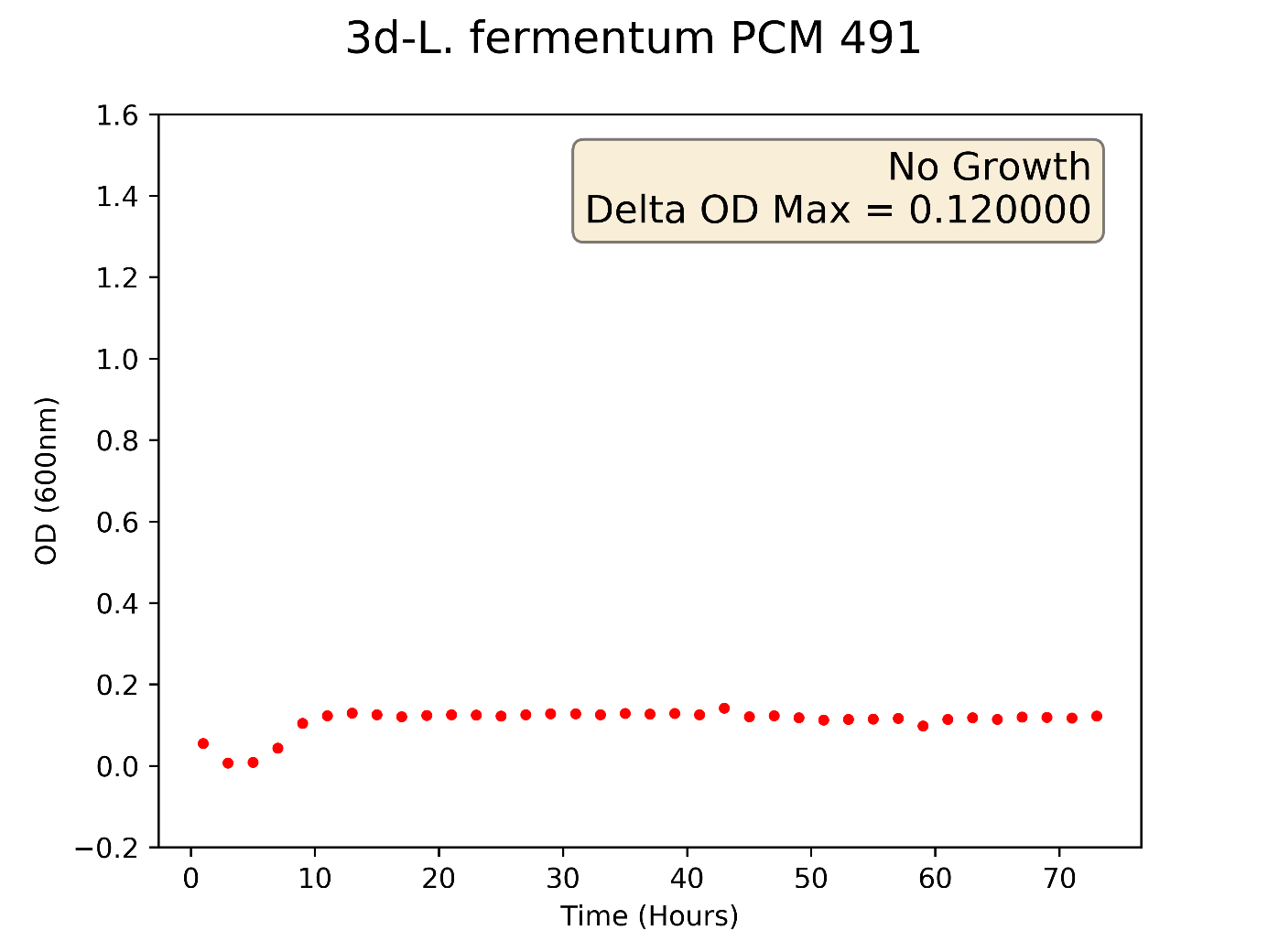


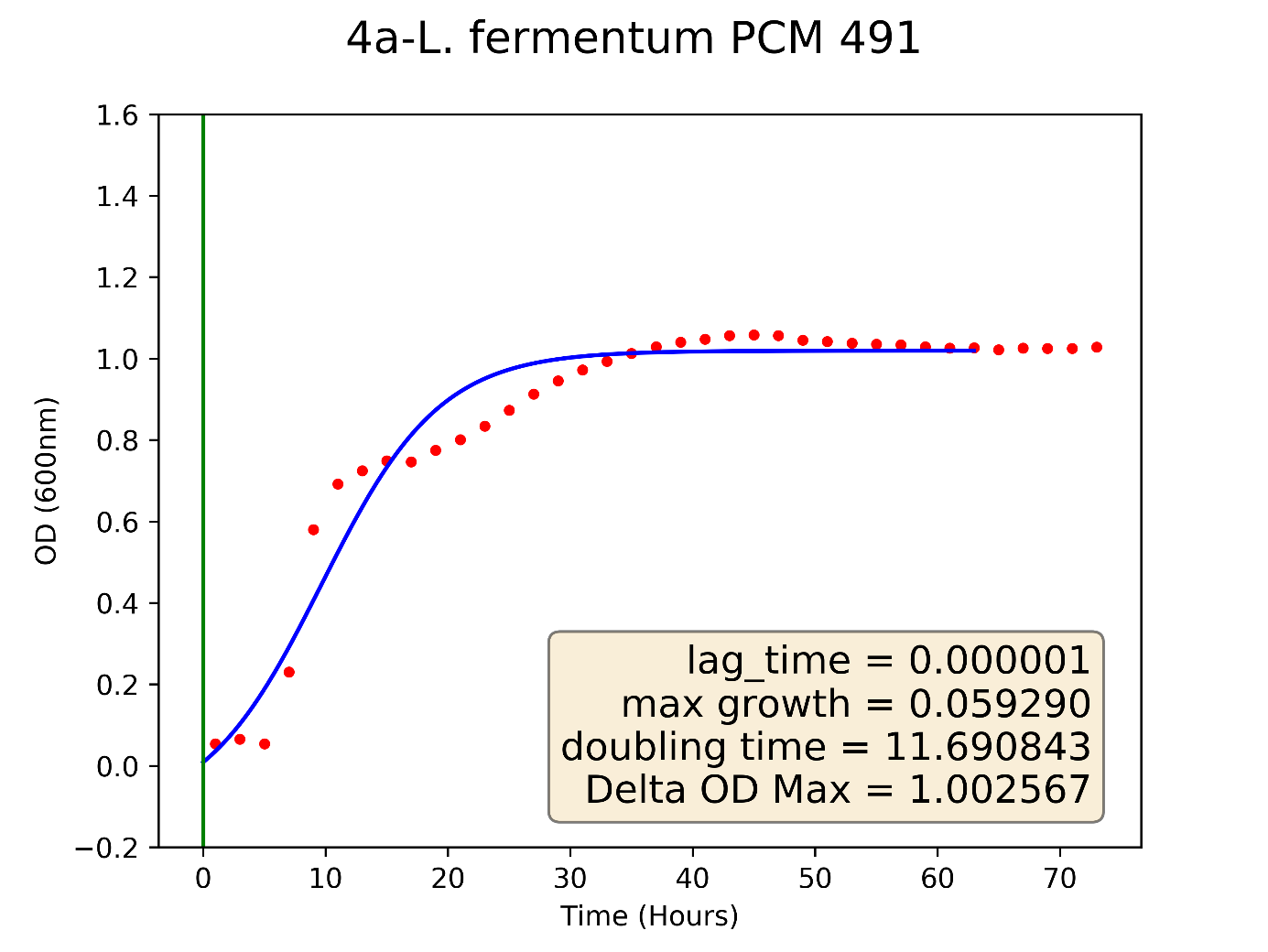


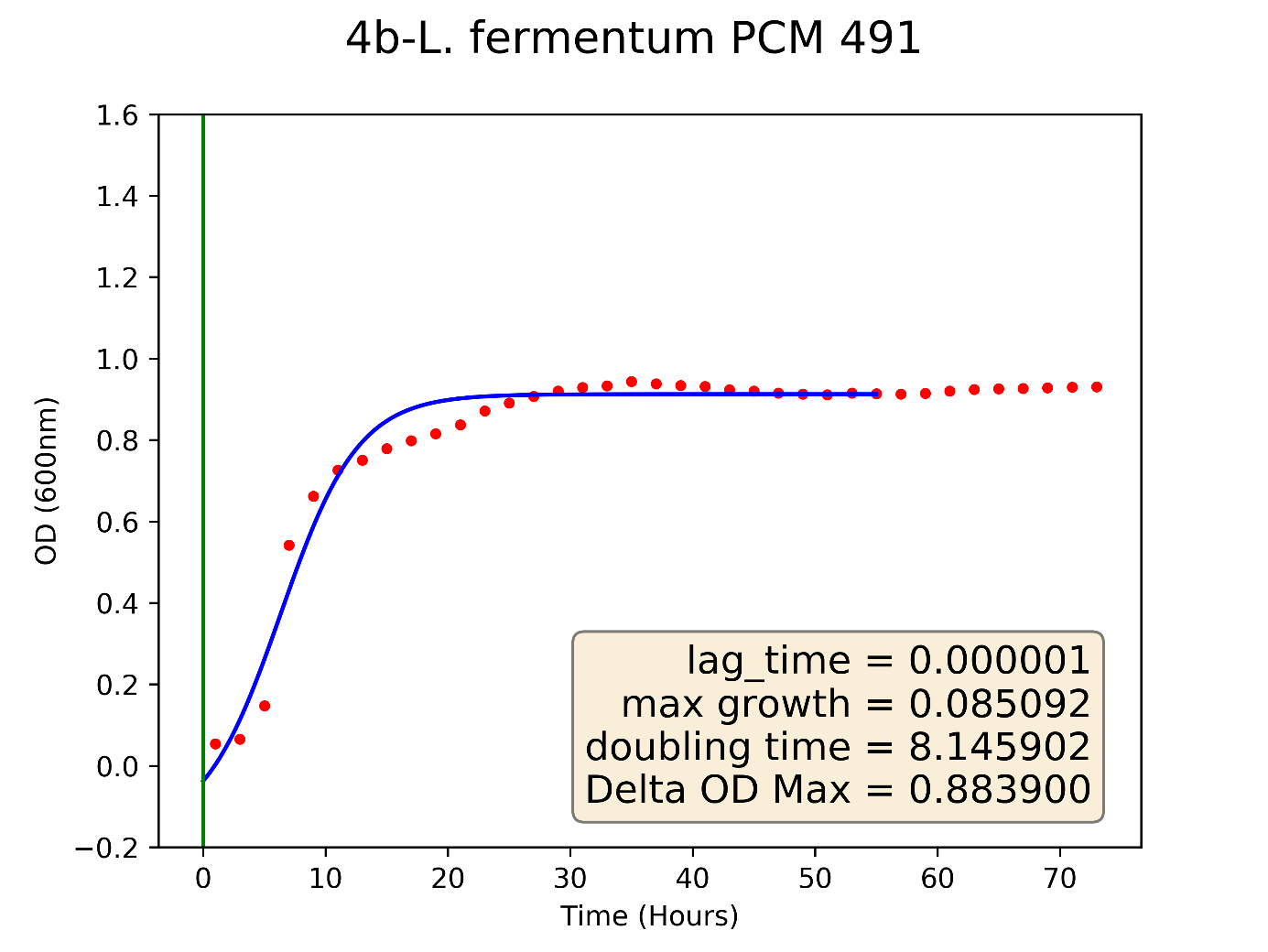


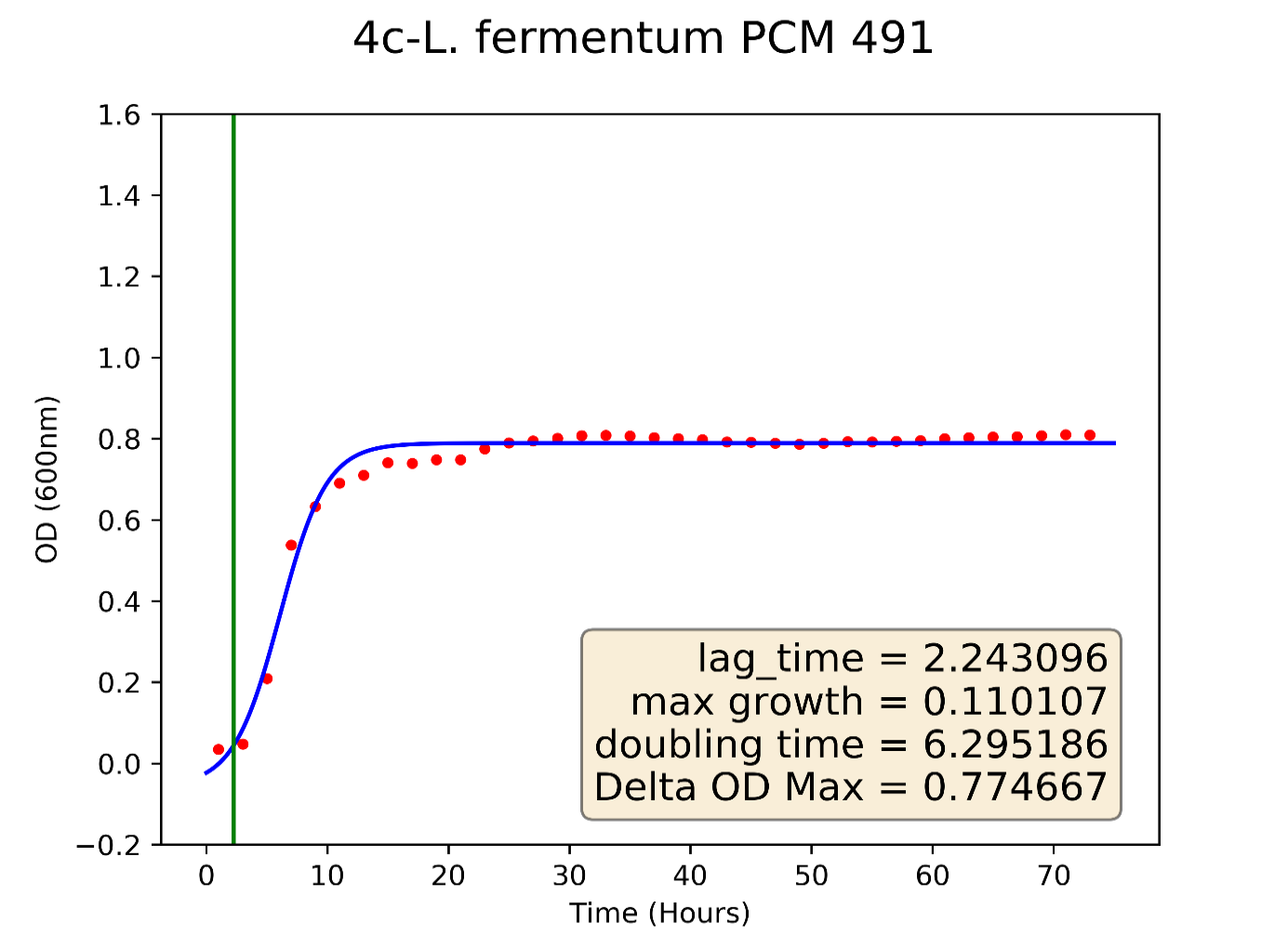


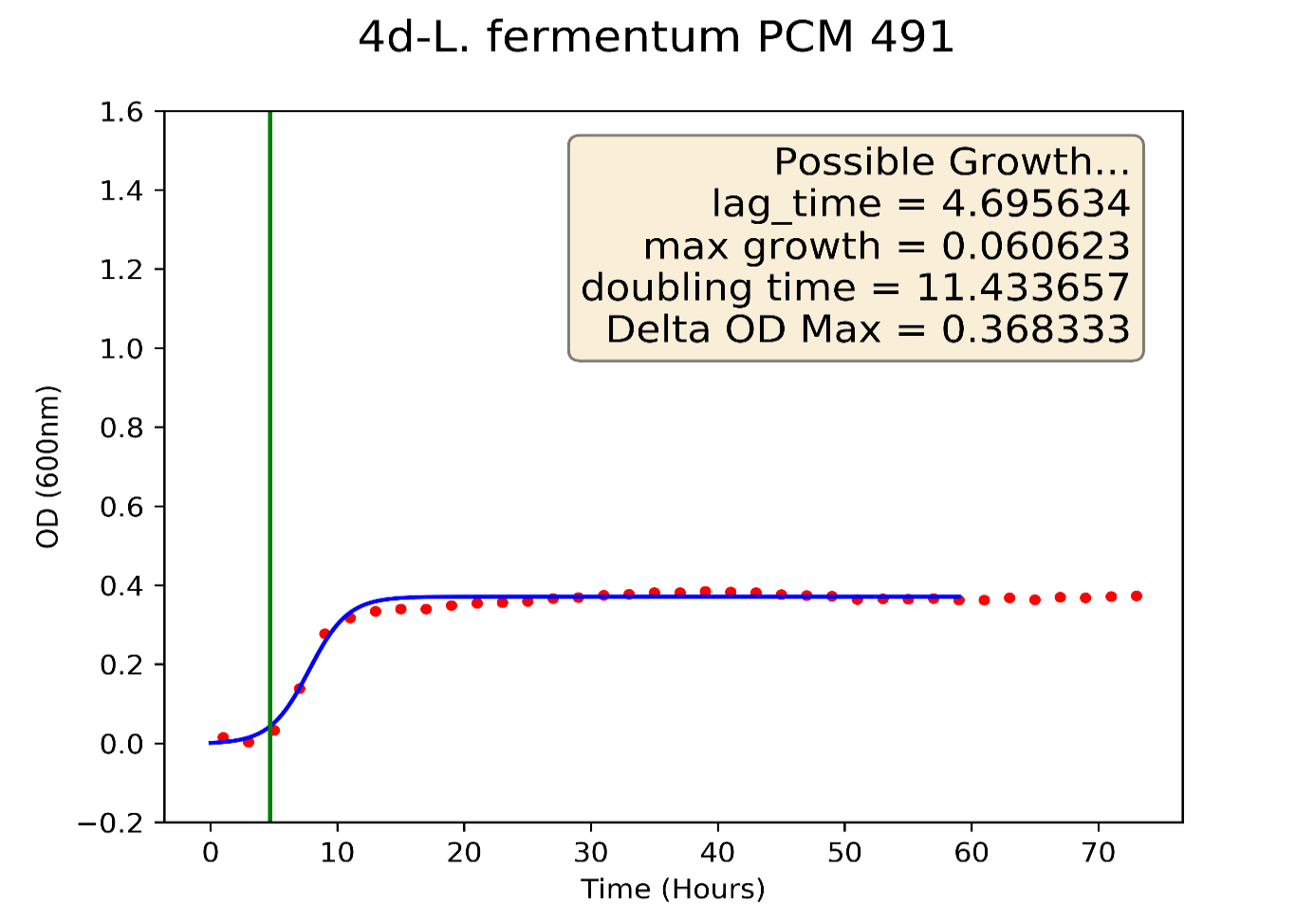


**Figure. S8.** Regression curves of selected data. *L. fermentum* (1, 2, 3, 4 – types of TiO_2_; a, b, c, d – concentration of TiO_2_ : 60, 150, 300 and 600 mg/L).
